# Pharmacokinetics of pirfenidone after topical administration in rabbit eye

**Published:** 2011-08-13

**Authors:** Guoying Sun, Xianchai Lin, Hua Zhong, Yangfan Yang, Xuan Qiu, Chengtian Ye, Kaili Wu, Minbin Yu

**Affiliations:** State Key Laboratory of Ophthalmology, Zhongshan Ophthalmic Center, Sun Yat-sen University, Guangzhou, China

## Abstract

**Purpose:**

Pirfenidone (5-methyl-1-phenyl-2-[1H]-pyridone) is a new, broad-spectrum agent that has an inhibition effect on the proliferation, migration, and collagen contraction of human Tenon’s fibroblasts, and thus modulating the wound healing process of glaucoma filtering surgical site. This study investigated the pharmacokinetics of topically administered pirfenidone (0.5%) in rabbit eyes.

**Methods:**

Pirfenidone solution (50 μl) was instilled into the rabbit’s conjunctival sac. The rabbits were quickly sacrificed at 2, 5, 8, 10, 15, 20, 30, 60, 90, and 120 min after the administration and ocular tissues were obtained. The concentrations of pirfenidone in conjunctiva, sclera, cornea, aqueous humor, and vitreous were determined by high performance liquid chromatography.

**Results:**

After topical administration, there was wide distribution and fast clearance of pirfenidone among the various ocular tissues. The mean maximum concentrations (C_max_) of pirfenidone in cornea, conjunctiva, sclera, aqueous humor, and vitreous were 9.64 mg/g, 9.62 mg/g, 2.13 mg/g, 34.88 mg/l and 0.52 mg/l, respectively. The half-life for these tissues was 18.26, 34.16, 15.71, 70.91, and 39.48 min, respectively.

**Conclusions:**

Measurable concentrations of pirfenidone are achieved in ocular tissues after topical application in rabbit model. Topical administration of pirfenidone may be an effective approach for modulation of wound healing responses in glaucoma filtration surgical site.

## Introduction

Glaucoma is the second leading cause of blindness worldwide [1]. Elevated intraocular pressure (IOP) is considered as the most consistent risk factor for glaucoma [2], although the pathogenesis of glaucomatous optic neuropathy remains unclear. Glaucoma filtration surgery such as trabeculectomy remains the mainstream of treatment [3]. Specifically, trabeculectomy offers an IOP-lowering effect by creating an outflow pathway from anterior chamber to subconjunctival spaces [4]. One of the important reasons for the failure of this surgery is scarring at filtering surgical site, which could block the aqueous humor outflow [5]. To address this problem, many anti-fibrotic products (e.g., 5-fluorouracil and mitomycin C) have been developed [4]. However, the administration of these agents has been limited by their toxicity and unappreciated complications (e.g., bleb leakage, hypotonous maculopathy, and infectious endophthalmitis) [6-11].

Pirfenidone (5-methyl-1-phenyl-2-[1H]-pyridone) is a new and broad-spectrum agent that has anti-fibrotic and anti-inflammatory effect in organs such as lung [12,13], liver [14], and kidney [15]. In a recent phase III multi-national clinical trial, pirfenidone has been shown to have beneficial effects for patients with various stages of idiopathic pulmonary fibrosis [16]. While the efficacy and safety of oral pirfenidone have been established in such specific diseases, limited data are available in its ophthalmic use.

Our group has previously shown that pirfenidone can prevent proliferation, migration and collagen contraction of human Tenon's fibroblasts in vitro [17]. We found that the inhibiting roles of pirfenidone on human Tenon's fibroblasts may be achieved by inhibiting mRNA and protein expression of transforming growth factor-β (TGF-β) isoforms [17]. Furthermore, we demonstrated that postoperative administration of 0.5% pirfenidone was associated with improved bleb survival in rabbit glaucoma surgery model [18]. In addition, pirfenidone has been shown to inhibit expression of tissue inhibitors of metalloproteinases-1 and it has anti-fibrotic effects on orbital fibroblasts from patients with thyroid-associated ophthalmopathy [19]. These findings warrant the potential therapeutic effects of pirfenidone for glaucoma filtration surgery and other fibrosis-related ocular diseases. To our knowledge, however, critical information regarding the pharmacokinetics of pirfenidone in various intraocular tissues has not been available. This study investigated the pharmacokinetics of 0.5% pirfenidone as a topically administered solution in eyes of live rabbits.

## Methods

### Animals

The study was conducted according to the ARVO Statement for the Use of Animal in Ophthalmic and Vision Research. The study was approved by the Welfare Committee of Animals in Zhongshan Ophthalmic Center, Guangzhou, China. Sixty Albino rabbits, weighting 2–2.5 kg each, were obtained from Medical Laboratory Animal Center, Guangdong Province. All animals were housed in clean cages at ambient temperature, and were acclimatized for at least one week before use.

### Drug preparations

Pirfenidone was provided by Sigma-Aldrich Co. (St. Louis, MO). Pirfenidone was dissolved in sterile water to achieve a final concentration of 0.005 g/ml (0.5% pirfenidone). The solutions were sterilized by the manufactory laboratory of Zhongshan Ophthalmic Center and then distributed equally into 60 sterile plastic eye-drop bottles, each assigned to one rabbit.

### Apparatus and chromatographic conditions

The high-performance liquid chromatograph (HPLC; Shimadzu, Kyoto, Japan) apparatus consisted of a Shimadzu LC-20AT separation module and an SPD-20A UV detector (Kyoto, Japan). Study was performed on a Luna 5-μm C18 column (150 mm×4.6 mm; Phenomenex, Torrance, CA). Samples were eluted from the column with an acetonitrile (A)-water (W) mobile phase (with a programming of 0~5 min: 35%A→65%A, 65%W→35%W; 5~5.5 min: 65%A→35%W; 5.5~8 min: 65%A→35%A, 35%W→65%W). Sample (20 μl) was injected into the column for analysis with a flow rate of 1 ml/min and the UV absorbance detector operated at 310 nm.

### Preparation of standard samples

A stock solution of pirfenidone was prepared and diluted with methanol to prepare working solutions at a variety of final concentrations (24.6, 12.3, 2.46, 1.23, and 0.62 mg/l). Working calibration curves for conjunctiva and aqueous humor were generated by adding a stock solution of pirfenidone to each of the blank matrices of aqueous humor or homogenates of conjunctival tissue. Ethyl 4-aminobenzoate (Sigma-Aldrich Co.) was mixed with methanol and used as internal standard (IS), with the concentration of 2.76 mg/l.

### Intra-day and inter-day precision

Intra-day and inter-day (on three different days) precision of measurement was evaluated by analyzing vitreous sample spiked with pirfenidone at the concentrations of 30.75, 0.62, and 0.077 mg/l. At each concentration, there were five repeated measurements and the precision was determined by relative standard deviation (RSD). A RSD <15% was considered acceptable. The extraction recovery of pirfenidone was measured by comparing the peak height of the extracted pirfenidone with that of the un-extracted standard sample. To prepare extracted samples, vitreous (0.1 ml) were separated and homogenized with 0.5 ml methanol. The supernatants were extracted and mixed with 50 μl pirfenidone and 50 μl IS. Each mixture was centrifuged at 15,294× g for 5 min. Supernatants (20 μl) were used as extracted samples.

### Stability study

After 3 cycles of freezing and thawing within 4 h, different concentrations of pirfenidone (30.75, 0.62, and 0.077 mg/l) were stored at room temperature (25 °C) for 24 h. The stability of pirfenidone was determined by relative error. A relative error within ±15% was considered acceptable.

### Treatment protocol and tissue preparation

Sixty rabbits were divided into ten groups with the use of a computer-assisted evenly distributed random number list. One drop (50 μl) of 0.5% pirfenidone solution was instilled topically into one eye of the rabbits. One group of six rabbits was sacrificed at the following time points after the instillation of drug: 2, 5, 8, 10, 15, 20, 30, 60, 90, and 120 min. The eyes were enucleated and the tissue samples of conjunctiva, cornea, sclera, aqueous humor (0.1 ml) and vitreous (0.1 ml) were separated and homogenized with 0.5 ml methanol and 50 μl IS. Each mixture was centrifuged at 15,294× g for 5 min. The supernatants were extracted, evaporated by flowing Nitrogen, resolved with 0.1ml methanol, and then centrifuged at 15,294× g for 5 min. Supernatants (20 μl) were used for analysis.

### Pharmacokinetics analysis

The pharmacokinetics of pirfenidone in cornea, conjunctiva, sclera, aqueous humor, and vitreous after topical administration were determined by a non-compartmental model (WinNonlin Professional, ver. 5.0; Pharsight Co. Mountain View, CA). Areas under the curve (AUC) were determined by a linear-trapezoidal method and the terminal half-life (t_1/2_) was estimated by linear regression of the concentration time points.

## Results

### Chromatographic separation and selectivity

**Figure 1:**
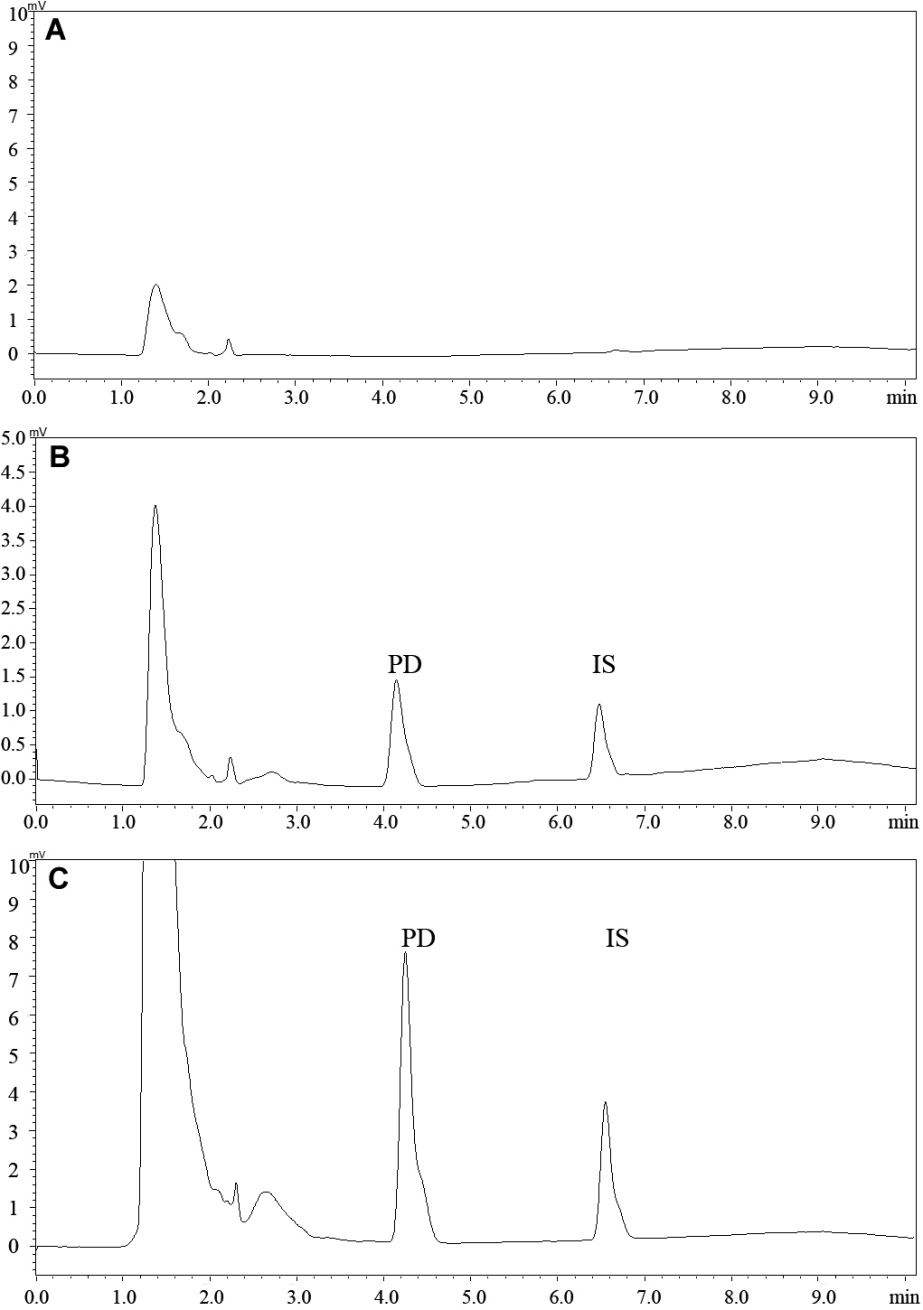
Chromatograms of cornea extracts. **A**: The chromatogram of blank cornea. **B**: The chromatogram of blank cornea spiked with pirfenidone and I.S. **C**: The chromatogram of cornea from the rabbit eye treated with pirfenidone and I.S.

The chromatograms of cornea are shown in Figure 1. Analysis on the blank samples showed that there were no interfering peaks in the chromatograms. The retention time for pirfenidone under the current conditions was around 4.3 min, and for internal standard it was about 6.5 min.

### Calibration linearity

Calibration curves for assay in conjunctiva and aqueous humor developed with peak area ratio of pirfenidone to internal standard versus drug concentration were linear over a wide range. The linear regression model was Y=0.0008+1.4312X (r=0.999, ranging from 0.02 to 6.15 mg/l) for conjunctiva, and Y=-0.0525+1.8425X (r=0.998, ranging from 0.08 to 30.75 mg/l) for aqueous humor.

### Intra-day and inter-day precision

At the concentrations of 30.75, 0.62, and 0.077 mg/l, the intra-day precision was 7.1, 5.5, and 8.4%, respectively, and the inter-day precision was 5.8, 3.6, and 6.9%, respectively. These results showed that the method was reproducible. For these concentrations, the extraction recoveries in aqueous humor were 97.2, 92.0, and 89.1%, respectively.

### Stability study

All the concentrations of pirfenidone (30.75, 0.62, and 0.077 mg/l) were stable at room temperature for 24 h. The concentrations of pirfenidone did not deviate from their original concentrations for more than 10%.

### Ocular pharmacokinetics

**Figure 2:**
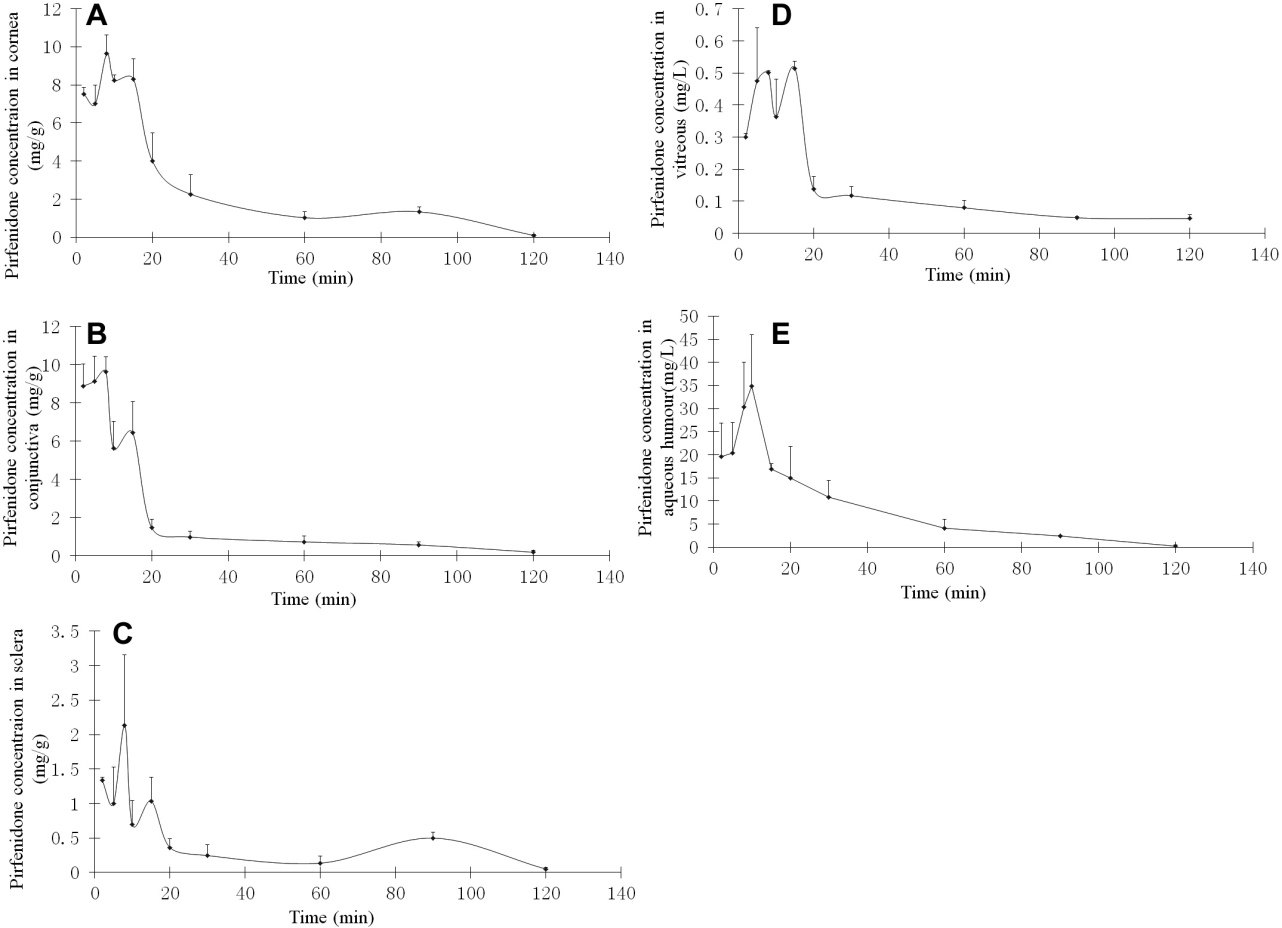
Pirfenidone concentration-time (C-t) profiles in ocular tissues. **A**-**E**: The C-t profiles obtained from cornea, conjunctiva, sclera, aqueous humor, and vitreous, respectively, after topical administration of pirfenidone.

Pirfenidone concentrations versus time profiles in various ocular tissues after topical administration are shown in Table 1 and Figure 2. The pharmacokinetic parameters are listed in Table 2. The concentration of pirfenidone peaked within 20 min among all the ocular tissues, and the distributions were fast. The C_max_ was 9.64 mg/g, 9.62 mg/g, 34.88 mg/l, 0.52 mg/l, and 2.13 mg/g for cornea, conjunctiva, aqueous humor, vitreous, and sclera, respectively.

**Table 1:** Ocular tissue concentrations of 0.5% pirfenidone solution after its topical administration in rabbit eyes.

Time (min)	Cornea (mg/g; Mean±SD)	Conjunctiva (mg/g; Mean±SD)	Sclera (mg/g; Mean±SD)	Aqueous (mg/l; Mean±SD)	Vitreous (mg/l; Mean±SD)
2	7.53±0.35	8.87±1.17	1.34±0.05	19.59±7.28	0.30±0.01
5	7.02±0.96	9.14±1.32	1.00±0.53	20.39±6.54	0.47±0.17
8	9.64±0.99	9.62±0.81	2.13±1.02	30.29±9.69	0.50±0.01
10	8.25±0.28	5.63±1.40	0.69±0.35	34.88±11.10	0.36±0.12
15	8.32±1.06	6.43±1.61	1.04±0.34	16.92±1.18	0.51±0.02
20	4.00±1.47	1.46±0.44	0.36±0.13	15.02±6.74	0.14±0.04
30	2.26±1.03	0.98±0.31	0.25±0.16	10.79±3.72	0.12±0.03
60	1.03±0.33	0.73±0.32	0.14±0.10	4.06±2.03	0.08±0.02
90	1.34±0.25	0.57±0.17	0.50±0.09	2.43±0.17	0.05±0.00
120	0.10±0.04	0.19±0.11	0.05±0.03	0.271±0.16	0.05±0.01

SD=standard deviation.

**Table 2:** Pharmacokinetic parameters in ocular tissues after topical administration of 0.5% pirfenidone solution in rabbit eyes.

**Pharmacokinetic parameters**	**Cornea**	**Conjunctiva**	**Sclera**	**Aqueous humor**	**Vitreous**
Tmax (min)	8	8	8	10	15
Cmax (mg/l)*	9.64*	9.62*	2.13*	34.88	0.52
AUC0-t (min•mg/l)	282.03	197.70	46.65	919.84	15.17
AUC0-∞ (min•mg/l)	284.58	206.91	49.34	925.99	19.88
T1/2 (min)	18.26	34.16	39.48	15.71	70.91
Lambda_z (1/min•10^–1^)	0.38	0.20	0.18	0.44	0.10

Cmax=observed maximum concentration; Tmax=time to Cmax; AUC0-t=area under the curve until the last measurable concentration; AUC0-∞=area under the curve from 0 to infinity; T1/2=Elimination half-life; and Lambda_z=First order rate constant associated with the terminal (log-linear) portion of the curve. *The concentration of pirfenidone in cornea, conjunctiva, and sclera is determined as milligrams per gram of tissue.

## Discussion

Pirfenidone has shown its anti-inflammatory and anti-fibrotic effect in in vitro and in vivo models of pulmonary and renal fibrosis [16]. Pirfenidone has shown its excellent oral or intravenous absorption and its pharmacokinetic behavior has been studied in mice [20], beagle dog [21], horse [22], sheep [23], and human [24-26]. In several large-scale phase III clinical trials, oral pirfenidone was safe and generally well tolerated [16,27]. The most frequent side effects included photosensitivity rash, nausea, dyspepsia, and dizziness [16,27].

Pirfenidone is known to inhibit a variety of cytokines in wound healing process. These include TGF-β [28], connective tissue growth factor (CTGF) [15], platelet-derived growth factors (PDGF) [29], and tumor necrosis factor-alpha (TNF-α) [30]. Our group has previously shown that pirfenidone had inhibiting effect on human Tenon’s fibroblasts and reduced wound healing response in glaucoma filtering site in rabbit model [17]. The in vitro study showed that the inhibiting effect of pirfenidone on human Tenon’s fibroblast started at the concentration of 0.15mg/ml and went into plateau at the concentration of 0.3 mg/ml [17]. Furthermore, both slit-lamp observation and histological analysis showed that topical administration of 0.1% and 0.5% pirfenidone had no apparent toxicity in cornea, ciliary body, and retina, with no apparent irritation to rabbit eye [18].

In this study, we characterized the pharmacokinetic profile of 0.5% pirfenidone after its topical administration in rabbit eyes. Pirfenidone in ocular tissues exhibited short-terminal half-lives (18 to 72 min). This rapid turnover is not unexpected and is very similar to the pharmacokinetics of many other ophthalmic agents such as latanoprost (t_1/2_ in the aqueous humor of rabbit=3 h) [31], timolol (t_1/2_=1.1 h) [32], and carteolol (t_1/2_=1.5 h) [33]. Topical ocular drug administration usually allows only a short contact time on the ocular surface. The duration of drug action may be prolonged by formulation design (e.g., gelifying formulations, ointments, inserts).

We were primarily interested in the 0.5% pirfenidone, as our previous study has shown that this dosage had a significant effect in inhibiting wound healing response in glaucoma filtering site in rabbit model [18]. It should be noted that the behavior of pirfenidone in human eyes may differ from that in rabbit eyes, given that the rabbit vitreous volume is smaller, and the retina is thinner, than those in human eyes. The uptake of pirfenidone in the conjunctiva was very quick, with the concentration peaking after 8 min (9.62 mg/ml) and diminishing to 0.19 mg/ml after 2 h. Therefore, if we assume that the in vitro environment and in vivo condition are similar, an initial topical administration of 0.5% pirfenidone may have a 2-h anti-fibrotic effect on glaucoma filtering region.

Given that pirfenidone has a broad spectrum of anti-fibrotic effect, further studies are needed to investigate whether pirfenidone could offer its potential therapeutic effect in fibrosis-related ocular diseases such as proliferative diabetic retinopathy and corneal wound healing response. These are currently under planning. Our study showed that pirfenidone could be internalized into conjunctiva, aqueous humor, vitreous, and sclera, in addition to cornea. The concentration of pirfenidone in vitreous was lower than in the cornea, but the concentration in aqueous humor was fourfold at 8 min and threefold at 120 min higher than in cornea. If pirfenidone is useful for the modulation of corneal wound response and proliferative diabetic retinopathy, it would become necessary to characterize its ocular concentration after subconjunctival and intravitreal administration.

In summary, we show that topical application of pirfenidone should be studied further as a modulating method for wound healing response after glaucoma filtering surgery. Measurable concentrations of pirfenidone are achieved in various ocular tissues. Formulation design may be needed to prolong the duration of drug action. Because of its broad spectrum of activity, pirfenidone has a potential for the modulation of other ocular wound healing responses as well.
